# Response to the Letter to the Editor Entitled “Optimizing Furosemide Use in Advanced CKD: Balancing Risks and Benefits”

**DOI:** 10.1016/j.ekir.2025.05.048

**Published:** 2025-06-03

**Authors:** M. Costes-Albrespic, N. Alencar de Pinho, Z.A. Massy, S. Liabeuf

**Affiliations:** 1 Clinical Epidemiology Team, Centre for Research in Epidemiology and Population Health, Inserm U1018, Paris-Saclay University, Versailles Saint-Quentin University, Villejuif, France; 2 Association pour l'Utilisation du Rein Artificiel, Paris, France; 3 Nephrology Department, Ambroise Paré University Hospital, APHP, Boulogne-Billancourt, Paris; 4 Pharmaco-epidemiology Unit, Department of Clinical Pharmacology, Amiens-Picardie University Medical Centre, Amiens, France; 5 MP3CV Laboratory, Jules Verne University of Picardie, Amiens, France

The Author Replies:

We thank T. Hannedouche and M. Ingwiller for their letter^1^ and J.-P. Bertocchio for his editorial,^2^ both of which highlight important aspects of the risk-benefit balance for furosemide in patients with chronic kidney disease and provide thoughtful insights on the basis of our results.^3^ Hannedouche, Ingwiller, and Bertocchio discuss possible clinical outcomes of loop diuretic use in patients who initiate dialysis. In particular, Ingwiller *et al.*^4^ have described an elevated likelihood of death and hospital admission in patients who received furosemide during more than 80% of their follow-up (as assessed by linkage to reimbursement information). Although the daily dose of furosemide could not be determined, it is noteworthy that nearly 80% of patients were supplied with 500 mg pills. However, possible residual confounding and the lead time bias inherent to the study design mean that causal inferences must be drawn with caution.

In contrast to Ingwiller *et al.*’s study, our work focused on patients with stage 3 or 4 chronic kidney disease and investigated the association between furosemide prescription and serum concentrations of free protein-bound uremic toxins.^3^ We found that only patients prescribed furosemide at a dose level > 120 mg (19% of the study cohort) had significantly higher serum protein-bound uremic toxin concentrations. Despite the limitations primarily associated with the observational nature of our study, these new results might constitute a bridge between Ingwiller *et al.*’s findings and our previous work on the adverse outcomes associated with high serum protein-bound uremic toxin levels.^5^ It would be useful to assess the effect of furosemide on serum protein-bound uremic toxin levels in patients on dialysis.

A more personalized approach is required for evidence-based medicine in general and loop diuretic therapy in particular. When prescribing furosemide, the physician must carefully assess the safety considerations, especially when alternative medications are available. This is particularly important for furosemide prescriptions at a high dose level and for long-term treatment. Along with the well-known adverse events associated with furosemide (i.e., ototoxicity and hypokalemia), a new potential risk—elevated uremic toxin levels—should also be considered.
